# The Effect of Different Laser Irradiation on Cyclophosphamide-Induced Leucopenia in Rats

**DOI:** 10.1155/2014/756406

**Published:** 2014-05-14

**Authors:** Jizhong Zhao, Ke Cheng, Haiping Deng, Ling Zhao, Lanlan Liu, Menghu Guo, Haimeng Zhang, Xueyong Shen

**Affiliations:** Acupuncture & Tuina College, Shanghai University of Traditional Chinese Medicine, Shanghai 201203, China

## Abstract

*Objective*. To assess the effect of different lasers on cyclophosphamide- (CTX-) induced leucopenia in rats. *Methods*. 11 rats were normal control and 55 rats were injected with a dose of 80 mg/kg CTX for the first time and 40 mg/kg on the 6th and the 11th days to establish a leucopenia model. Rats of the irradiation groups received a 5-minute laser irradiation with either single 10.6 **μ**m or 650 nm laser or alternatively 10.6 **μ**m–650 nm laser irradiation, besides a sham treatment on acupoint Dazhui (DU 14) and acupoint Zusanli (ST 36) of both sides, 8 times for 16 days. Normal and model control group received no treatment. *Results*. On day 16 after the first CTX injection, the WBC counts from all the laser irradiation groups were significantly higher than those from the model control and the sham group (*P* < 0.05), while there were no significant differences compared with the normal control (*P* > 0.05). The TI of 10.6 **μ**m–650 nm laser irradiation group was significantly higher than that of the model control group (*P* < 0.05). *Conclusions*. The single and combined 10.6 **μ**m and 650 nm laser irradiation on ST36 and DU14 accelerated the recovery of the WBC count in the rats with leucopenia.

## 1. Introduction

Leucopenia, a disease with a white blood cell (WBC) number consistently lower than 4.0 × 10^9^/L in the peripheral blood, can be classified into primary and secondary leucopenia. The main secondary cause of leucopenia is bone marrow suppression that resulted from antitumor drugs. Cancer chemotherapy drugs have long been considered immune suppressive [1]. Bone marrow suppression can severely influence the effect of chemotherapy as well as quality of life after chemotherapy. As a main approach for antitumor therapy, chemotherapy can improve the pathological state of the patients. However, its side effects, such as bone marrow suppression, also bring harm to the patients' physical, mental, and economical states. Clinical studies showed that acupuncture and moxibustion associated with relief of side effects and increase of leukocyte in patients with leucopenia induced by chemotherapy. However, the evidence was of low quality [2–5]. Animal studies found that electroacupuncture and acupoint injection showed benefit on bone marrow suppression after chemotherapy, through protection of hematopoietic function, improvement of WBC counts in the peripheral blood, and enhancement of immune function [6, 7]. However, needling acupuncture might result in some degrees of discomfort, such as pain, while moxibustion produces smog, which could be irritant and harmful to the health of both the practitioners and patients [8–10]. These factors limited the application of traditional acupuncture in the clinical use. Laser acupuncture/moxibustion is a good substitute of traditional acupuncture/moxibustion and has been researched for more than 20 years [11]. Laser acupuncture/moxibustion causes no skin penetration and smoke and is easier to practice and regulate. Our previous research found that the CO_2_ laser with the wavelength of 10.6 *μ*m and semiconductor laser with the wavelength of 650 nm have shown some benefit for pituitrin-induced bradycardia and knee osteoarthritis [12, 13]. The purpose of this study was to explore the effect and the mechanism of acupoint irradiation with the single 10.6 *μ*m CO_2_ laser, the single 650 nm semiconductor laser, and the combination of the two lasers in cyclophosphamide- (CTX-) induced leucopenia.

## 2. Materials

### 2.1. Animals

A total of 66 healthy adult male SD rats were supplied by Animal Experiment Center of Shanghai Traditional Chinese Medicine University, with body weight of 200 ± 20 g and normal white blood cell (WBC) count. The license number for animal manufacture was SCXK (HU) 2009-0018, bought from Shanghai Xipuer-Bikai Lab Animal Co., Ltd. The license number for the use of experimental facilities in Shanghai University of Traditional Chinese Medicine was SCXK (HU) 2009-0069.

### 2.2. Reagents

The CTX (0.2 g) used to induce leucopenia was bought from Jiangsu Hengrui Pharmaceutical Incorporated Company. The manufacturing lot number for it was H32020857.

### 2.3. Apparatus

The CO_2_ laser acupuncture apparatus (SX10-C1) and the 650 nm semiconductor laser acupuncture apparatus were produced by Shanghai Wanqi Photoelectric Technology Co., Ltd. The wavelength of the CO_2_ laser was 10.6 *μ*m and its output power was 55 ± 5 mw. The wavelength of the semiconductor laser was 650 nm and its output power was 36 mw with a diameter of light spot of 1 mm [12]. ANI full-automatic animal blood cell analyzer (XFA6030) was bought from Nanjing Pulang Medical Equipment Co., Ltd.

## 3. Methods

### 3.1. Establishment of Rat Leucopenia Model

All animals were raised and bred in the same standard environment, with the room temperature of (24 ± 1)°C and humidity of 50–70%. An improved method to establish animal model for leucopenia was used [14, 15]. The rats were weighed and CTX was used for intraperitoneal injection in a body weight-dependent dose manner. A total of 80 mg/kg of CTX was injected for the first time and 40 mg/kg on the 6th and the 11th days.

### 3.2. Selection and Location of Acupoints

Acupoint Dazhui (DU 14) and acupoint Zusanli (ST 36) of both sides were selected on the basis of TCM theory as well as the previous clinical studies [16]. Acupoints were located referring to standards described in “Experimental Acupuncture Science” [17]. We located Zusanli at posterolateral knee joint, about 5 mm underneath the fibulae capitulum, and Dazhui between the spinous process of the 7th cervical vertebra and the 1st thoracic vertebra, in the middle of the back [17].

### 3.3. Animal Grouping

Rats were numbered according to their body weight. A total of 66 rats were randomly allocated into six groups with a random number produced by SPSS18.0. These groups included normal control group, model control group, sham laser irradiation group, 10.6 *μ*m CO_2_ laser irradiation group, 650 nm semiconductor laser irradiation group, and combined 10.6 *μ*m–650 nm laser irradiation group. With the exception of normal control group, the rats in the other five groups were induced by leucopenia with CTX. After inducing leucopenia, the 3 true laser groups were irradiated on the acupoint Dazhui (DU 14) and bilateral acupoint Zusanli (ST 36) with 10.6 *μ*m CO_2_ laser, 650 nm semiconductor laser, and combined 10.6 *μ*m–650 nm laser, respectively. Each acupoint received irradiation for 5 minutes (min) every other day, with a total of 8 sessions of irradiation. The sham laser irradiation group was treated the same way as true laser groups, but without laser output.

### 3.4. Sample Collection and Detection

#### 3.4.1. Detection of WBC Counts in the Peripheral Blood Collected from Tail End of the Rats

Rat tail was cut and blood was collected into tubes with anticoagulant one day before as well as 1, 4, 15, and 16 days after the CTX injection. Animal blood cell analyzer was used to count the blood cell numbers. WBC counts were also recorded.

#### 3.4.2. Calculation of Thymus Index (TI)

Thymus gland was excised from each rat on the 16th day after the first injection of CTX, washed with physiological saline, and weighed using a balance with 0.0001 accuracy. The TI was calculated with the formula TI = (index of thymus weight/body weight) × 10.

### 3.5. Statistical Analysis

The SPSS18.0 software was used for statistical analysis. The outcome data was expressed as mean ± standard deviation (mean ± SD) when the data accords with normal distribution. One-way ANOVA was used for comparison between groups and ANOVA for repeated measurement method was used in comparison within groups. The TI was analyzed with one-way ANOVA and the differences were considered statistically significant when the *P* was less than 0.05.

## 4. Results

### 4.1. Establishment of Rat Leucopenia Model

Before the first CTX injection, WBC counts of the rats in all groups were normal showing no statistically significant differences (*P* > 0.05). After CTX injection, the WBC counts in the other 5 groups significantly decreased compared with the normal control group (*P* < 0.05), while there were no statistically significant differences among the 5 groups (*P* > 0.05). These results indicated that we successfully established the leucopenia model in rat (Table 1). There was one rat that died after CTX injection in each of the model control, sham irradiation, 10.6 *μ*m laser irradiation group, and 10.6 *μ*m– 650 nm irradiation group.

### 4.2. Changes of WBC Counts after Laser Irradiation

Results showed that WBC counts decreased after the first CTX injection and then rose at day 16. On the first and fourth days, WBC counts were significantly lower in all the other 5 groups compared to that of the normal control group (*P* < 0.01). Sixteen days after first CTX injection, the WBC counts returned to normal level in the 3 true laser irradiation groups with no statistically significant difference compared to that in the normal control group (*P* > 0.05). WBC counts were still lower in the model control and sham irradiation groups as compared to that in the normal control group (*P* < 0.05) (Table 1).

Sixteen days after the first CTX injection, the WBC counts were statistically significantly higher in each group treated with laser irradiation as compared to that in the model control group (*P* < 0.05) and sham irradiation group (*P* < 0.05). The WBC counts in the sham irradiation and model control groups showed no statistically significant difference at any of the time points (*P* > 0.05).

### 4.3. Thymus Index

Sixteen days after the first CTX injection, the TI in each group with laser irradiation was significantly lower than that of the normal control group (*P* < 0.01), while the TI in the 10.6 *μ*m–650 nm laser group was statistically significantly higher than that of the model control group (*P* < 0.05) (Table 2).

## 5. Discussion

Our result indicated that WBC counts in groups with irradiation of 10.6 *μ*m CO_2_ laser, 650 nm semiconductor laser, and 10.6 *μ*m–650 nm laser recovered more quickly than those in the sham irradiation and model control groups. Since there was no difference between the WBC counts in the sham irradiation and model control groups, the effect of the factors other than laser acupuncture stimulation on experimental results, such as the fixation of rats, might be excluded. This finding was similar to our previous pilot study, in which we found similar therapeutic effect of 10.6 *μ*m CO_2_ laser and 650 nm semiconductor laser for the CTX-induced leucopenia in rat.

Thymus gland reflects the state of the immune function, which might be suppressed by chemotherapy. Results showed that only the combination of 10.6 *μ*m–650 nm laser irradiation improved the TI of the leucopenia model rats, while neither 10.6 *μ*m laser nor 650 nm laser alone improved the TI, indicating that the combination of both lasers is necessary to improve the immune function, which is in accordance with the previous research findings [18].

In the view of traditional Chinese medicine, leucopenia belongs to the category of consumptive disease and blood deficiency. The chemotherapy drugs are normally working well to treat cancerous cell; however, their toxicity is harmful to Qi and blood, as well as the function of Zang and Fu organs. Stomach is a reservoir of food and drink as well as a source of Qi and blood. Zusanli (ST 36), located on the stomach meridian, can regulate and tonify the spleen and stomach Qi, thus regulating and facilitating Qi and blood. Dazhui (Du 14), the crossing point of all Yang meridians on the Du meridian, can invigorate Yang Qi and blood. Both ST 36 and DU 14 are the most commonly used acupoints in treating leucopenia as reported [16].

A general method used for model establishment with the CTX is to inject animals only one time, lasting for 1 to 5 days according to the dose. We have used this method in our previous study and established a model for rat leucopenia, which lasted for 10 days [18]. After iterative trial and error, we finally chose the method of multiple injection of CTX at a low dose in order to establish the model for rat leucopenia. With this method, the established model lasted for about 16 days, which was considered as a relatively steady model that replicated leucopenia caused by clinical chemotherapy and was convenient for observation of curative effects.

The CO_2_ laser is a far-infrared light with the wavelength of 10.6 *μ*m, which can be absorbed by epidermis within 0.2 mm and can bring about a rapid, obvious, and abiding heat effect [19]. The 650 nm semiconductor laser can reach the deep epidermis, stimulate several sensors on the nerve ending at the acupoint, and result in effects similar to those of acupuncture. Our previous study on animals indicated that 10.6 *μ*m–650 nm laser irradiation on acupoint Neiguan had effect on pituitrin-induced bradycardia in rabbit, with CO_2_ laser playing the main role in this action [12].

In conclusion, irradiation with the 10.6 *μ*m laser, 650 nm laser, and combined 10.6 *μ*m–650 nm laser on certain acupoints boosted the recovery of WBC counts in the peripheral blood and improved the hematopoietic function of the rats with leucopenia. The combination of both lasers might be necessary to improve the immune function. This approach and its mechanism of action need further investigations.

## Figures and Tables

**Table 1:** WBC counts in rat peripheral blood (mean ± SD, ×10^9^).

Group name	Animal number	Before the first CTX injection	After the first CTX injection		
			1 d	4 d	16 d
Normal control	11	11.70 ± 4.36	7.72 ± 2.85	9.47 ± 3.9	11.17 ± 1.48
Model control	10	13.87 ± 3.12^1^	2.50 ± 0.69^2^	2.48 ± 2.01^2^	6.24 ± 3.37^2^
Sham irradiation	10	14.19 ± 2.94^1^	1.26 ± 0.61^2^	1.20 ± 0.54^2^	7.33 ± 4.17^2^
10.6 *μ*m–650 nm	10	11.25 ± 1.77^1^	0.76 ± 0.62^2^	1.35 ± 1.34^2^	10.01 ± 4.17^1,3,4^
650 nm	11	11.83 ± 2.06^1^	1.88 ± 0.61^2^	2.42 ± 0.95^2^	10.11 ± 3.11^1,3,4^
10.6 *μ*m	10	11.66 ± 2.17^1^	1.26 ± 0.47^2^	1.68 ± 1.10^2^	11.89 ± 5.56^1,3,4^

Compared with normal control: ^1^ *P* > 0.05, ^2^ *P* < 0.05; compared with model control: ^3^ *P* < 0.05; compared with sham irradiation: ^4^ *P* < 0.05.

**Table 2:** The TI of each group 16 days after model establishment.

Group name	Animal numbers	Weight (g)	TI
Normal control	11	307.36 ± 20.92	1.7637 ± 0.1257
Model control	10	240 ± 12.27	0.6327 ± 0.0610^1^
Sham irradiation	10	232.5 ± 17.46	0.8773 ± 0.0765^1^
10.6 *μ*m–650 nm	10	244.5 ± 19.03	1.1174 ± 0.2950^2^
650 nm	11	243.73 ± 19.92	0.9361 ± 0.0961^1^
10.6 *μ*m	10	242.1 ± 19.90	0.8668 ± 0.0902^1^

Compared with normal control group: ^1^ *P* < 0.05; compared with model control group: ^2^ *P* < 0.05.
